# Acute Exacerbation of Previously Unrecognized Interstitial Lung Disease in a Functionally Independent Nonagenarian: A Case Report

**DOI:** 10.7759/cureus.113798

**Published:** 2026-08-01

**Authors:** Rei Hosaka, Taichi Fujimori, Ryuichi Ohta, Chiaki Sano

**Affiliations:** 1 Faculty of Medicine, The Jikei University School of Medicine, Tokyo, JPN; 2 Community Care, Unnan City Hospital, Unnan, JPN; 3 Community Medicine Management, Shimane University Faculty of Medicine, Izumo, JPN

**Keywords:** acute exacerbation of interstitial lung disease, frailty, healthcare-seeking behavior, interstitial lung disease, older adults

## Abstract

Acute exacerbation of interstitial lung disease (AE-ILD) is associated with high mortality and a poor prognosis. Particularly in the oldest-old population, comprehensive assessment, including factors contributing to delays in seeking medical care and decisions regarding treatment eligibility, is clinically important. A 92-year-old man, who had been independent in activities of daily living (ADLs), ambulated with a cane, and lived at home before admission, was treated for presumed pneumonia at a local clinic because of a cough. However, his symptoms did not improve, and he was referred to our hospital because of worsening dyspnea. Chest computed tomography (CT) revealed bilateral ground-glass opacities and pre-existing pulmonary fibrosis. He was clinically diagnosed with previously undiagnosed interstitial lung disease (ILD) complicated by bacterial pneumonia, and treatment with antibiotics and corticosteroids was initiated. His respiratory status initially improved; however, chest CT performed on hospital day 5 showed worsening bilateral ground-glass opacities. On hospital day 6, his respiratory status rapidly deteriorated, leading to a diagnosis of AE-ILD. Despite treatment with corticosteroid pulse therapy and cyclophosphamide, he ultimately died. In this case, the delay in seeking medical care may have been related not only to the patient’s awareness of his disease but also to healthcare system factors, including the timing of referral to our hospital and the availability of continuous follow-up. Although the patient was 92 years old, his pre-admission ADLs were preserved; therefore, aggressive treatment was selected based on his functional status rather than chronological age alone. Nevertheless, this case suggests that AE-ILD can follow a rapidly progressive and fatal course even in patients with preserved ADLs. In the management of AE-ILD in the oldest-old population, the factors underlying delays in seeking medical care should be assessed from both patient- and healthcare system-related perspectives. Furthermore, treatment eligibility should not be determined solely on the basis of chronological age; individualized care based on a comprehensive assessment of ADLs and the patient’s overall clinical background is essential.

## Introduction

Interstitial lung disease (ILD) comprises a group of disorders characterized primarily by inflammation and fibrosis of the alveolar walls. Its incidence is particularly high among older adults, and acute exacerbations are known to be triggered by infections, medications, autoimmune mechanisms, and other factors [1,2]. Clinical manifestations include cough, dyspnea, and fever, and characteristic imaging findings include ground-glass opacities and reticulation on chest CT [1]. Diagnosis is made through a comprehensive assessment of the clinical course, imaging findings, and evaluation for the presence or absence of infection [1]. In addition to management of the underlying disease, corticosteroid pulse therapy and immunosuppressive agents, such as cyclophosphamide, may be considered during acute exacerbations. However, the prognosis remains poor, and particularly in older patients, determining the treatment strategy requires balancing treatment invasiveness with the patient’s clinical background [1,2].

The present case involved a 92-year-old man who presented with cough, dyspnea, and fever. After further evaluation, he was clinically diagnosed with previously undiagnosed ILD complicated by bacterial pneumonia and was treated with ceftriaxone and prednisolone. Incidentally detected fibrotic interstitial lung abnormalities are increasingly recognized as clinically important because they are associated with disease progression and warrant appropriate clinical evaluation and follow-up [3]. The clinical significance of this case lies not merely in the delay in presentation but in the convergence of several clinically relevant issues: previously unrecognized ILD despite pre-existing radiological abnormalities, delayed access to our hospital in the context of both patient- and healthcare system-related factors, and the challenge of determining treatment eligibility in a functionally independent nonagenarian who subsequently developed rapidly progressive acute exacerbation of interstitial lung disease (AE-ILD). This case therefore provides important clinical insights into healthcare-seeking behavior, regional healthcare coordination, early recognition and follow-up of underlying ILD, and individualized treatment decision-making based on functional status rather than chronological age alone.

## Case presentation

A 92-year-old man (height, 158 cm; weight, 53.1 kg) was independent in ADL, ambulated with a cane, and lived at home before admission. He had no medical history suggestive of immunodeficiency. He had experienced a chronic cough, predominantly at night, for some time but had not undergone further evaluation at a medical institution. His cough worsened one week before presentation. Four days before presentation, he was treated for presumed pneumonia at a local clinic with dimemorfan phosphate, L-carbocisteine, and clarithromycin; however, his symptoms did not improve. On the day of presentation, his dyspnea worsened with fever, and chest radiography suggested bilateral pneumonia. He was therefore referred to our hospital for further evaluation.

Physical examination

On presentation, his blood pressure was 139/74 mmHg, pulse rate was 86 beats/min, body temperature was 37.1 °C, respiratory rate was 24 breaths/min, and oxygen saturation was 90% on room air. No jugular venous distention was observed, and fine end-inspiratory crackles were heard bilaterally.

Investigations and diagnosis

**Figure 1:**
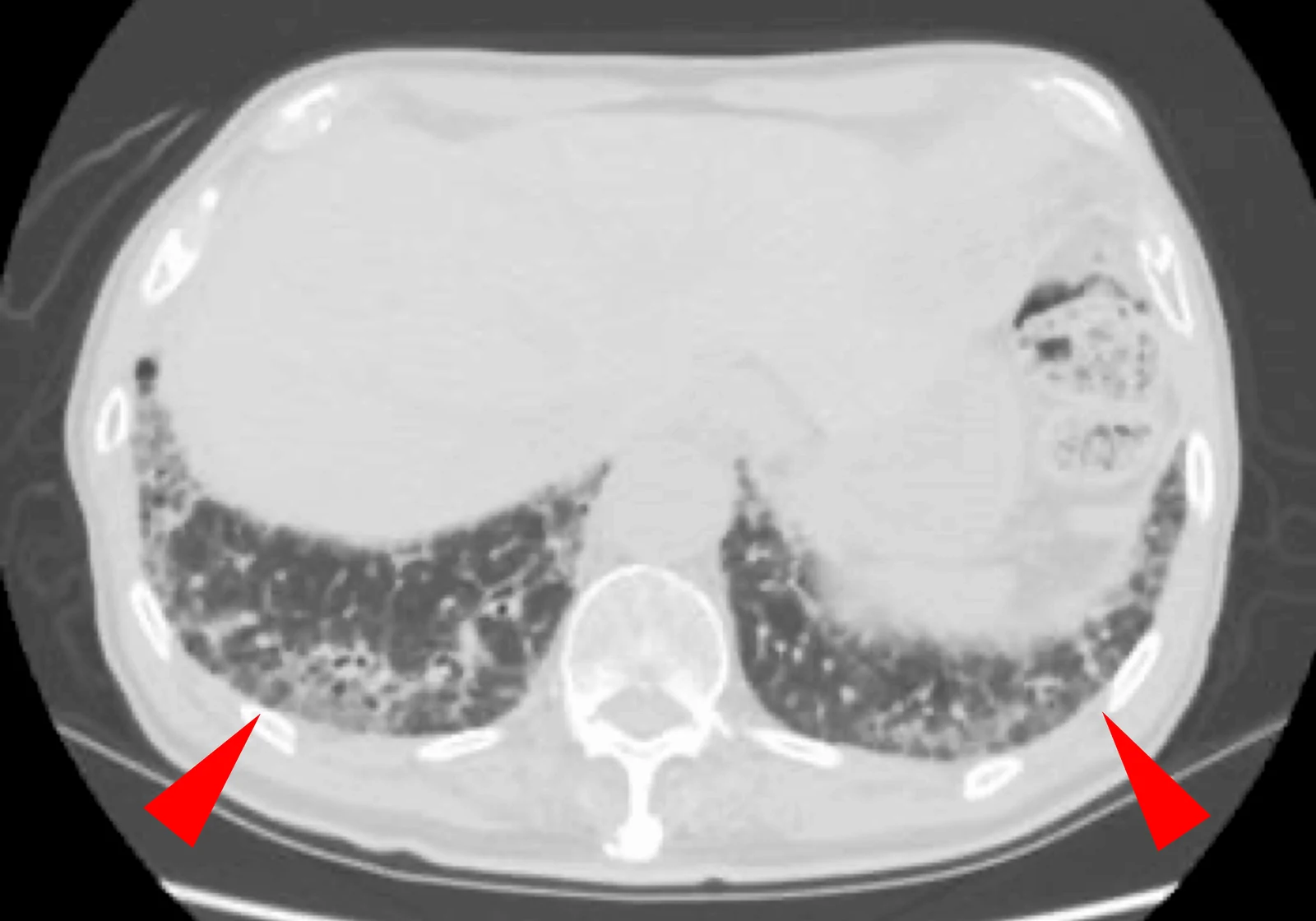
Chest computed tomography performed three years before admission Chest computed tomography demonstrating bilateral subpleural reticular opacities and fibrotic changes, highlighted by red arrowheads

**Figure 2:**
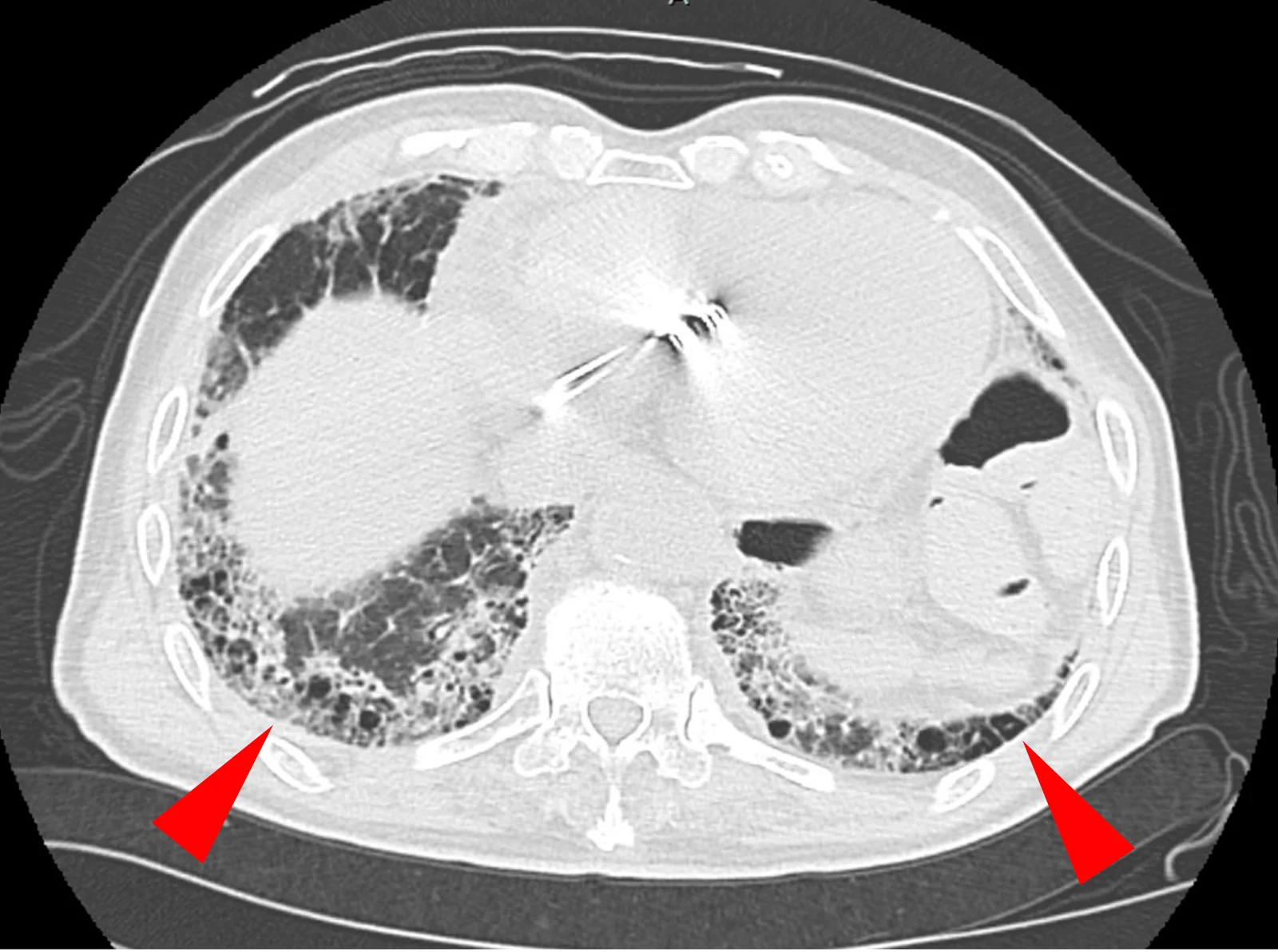
Chest computed tomography on admission Chest computed tomography demonstrating bilateral ground-glass opacities superimposed on pre-existing fibrotic changes, highlighted by red arrowheads

Chest radiography revealed scattered ground-glass opacities in both lungs. Initial laboratory tests showed elevated C-reactive protein and lactate dehydrogenase levels and a mildly elevated Krebs von den Lungen-6 level (Table 1). Autoantibody testing revealed no apparent connective tissue disease-associated autoantibodies (Table 2). Chest CT performed three years previously had shown only predominantly subpleural reticulation and fibrosis in the lower lungs (Figure 1). In contrast, chest CT on admission revealed new bilateral diffuse ground-glass opacities in addition to the pre-existing fibrosis (Figure 2). He was clinically diagnosed with previously undiagnosed ILD complicated by bacterial pneumonia. Despite these previous CT findings, he had never been diagnosed with ILD and had remained untreated.

**Table 1:** Initial laboratory data of the patient

Parameter	Level	Reference Range
White blood cells	8.2 × 10^3^/μL	3.5–9.1 × 10^3^/μL
Neutrophils	85.4%	44.0–72.0%
Lymphocytes	7.2%	18.0–59.0%
Hemoglobin	10.4 g/dL	11.3–15.2 g/dL
Hematocrit	30.3%	33.4–44.9%
Mean corpuscular volume	91.8 fL	79.0–100.0 fL
Platelets	29.8 × 10^4^/μL	13.0–36.9 × 10^4^/μL
Total protein	7.0 g/dL	6.5–8.3 g/dL
Albumin	2.7 g/dL	3.8–5.3 g/dL
Total bilirubin	0.9 mg/dL	0.2–1.2 mg/dL
Aspartate aminotransferase	38 IU/L	8–38 IU/L
Alanine aminotransferase	26 IU/L	4–43 IU/L
Lactate dehydrogenase	392 U/L	121–245 U/L
Blood urea nitrogen	28.7 mg/dL	8–20 mg/dL
Creatinine	0.65 mg/dL	0.40–1.10 mg/dL
Serum Na	126 mEq/L	135–150 mEq/L
Serum K	4.2 mEq/L	3.5–5.3 mEq/L
Serum Cl	91 mEq/L	98–110 mEq/L
Ferritin	183.1 ng/mL	14.4–303.7 ng/mL
CRP	9.89 mg/dL	<0.30 mg/dL
IgG	1553 mg/dL	870–1700 mg/dL
IgM	37 mg/dL	35–220 mg/dL
IgA	306 mg/dL	110–410 mg/dL
Creatine kinase	485 U/L	56–244 U/L
Sialylated carbohydrate antigen KL-6	505 U/mL	105.3–401.2 U/mL

**Table 2:** Autoantibody and interstitial lung disease-related serological findings

Parameter	Level	Reference Range
Antinuclear antibody	<1:40	<1:40
Homogeneous	<1:40	<1:40
Speckled	<1:40	<1:40
Nucleolar	<1:40	<1:40
Peripheral	<1:40	<1:40
Discrete speckled	<1:40	<1:40
Cytoplasmic	<1:40	<1:40
Myeloperoxidase anti-neutrophil cytoplasmic antibody (MPO-ANCA)	<1.0 U/mL	<3.5 U/mL
Rheumatoid factor	0 IU/mL	<15 IU/mL
Anti-cyclic citrullinated peptide antibody	<0.6 U/mL	<4.5 U/mL
Surfactant protein D	307 ng/mL	<110 ng/mL
Surfactant protein A	81.0 ng/mL	<43.8 ng/mL
Anti-aminoacyl-tRNA synthetase (anti-ARS) antibody	<5.0	<25.0
Anti-melanoma differentiation-associated gene 5 antibody	<4	<31

Treatment and clinical course

**Figure 3:**
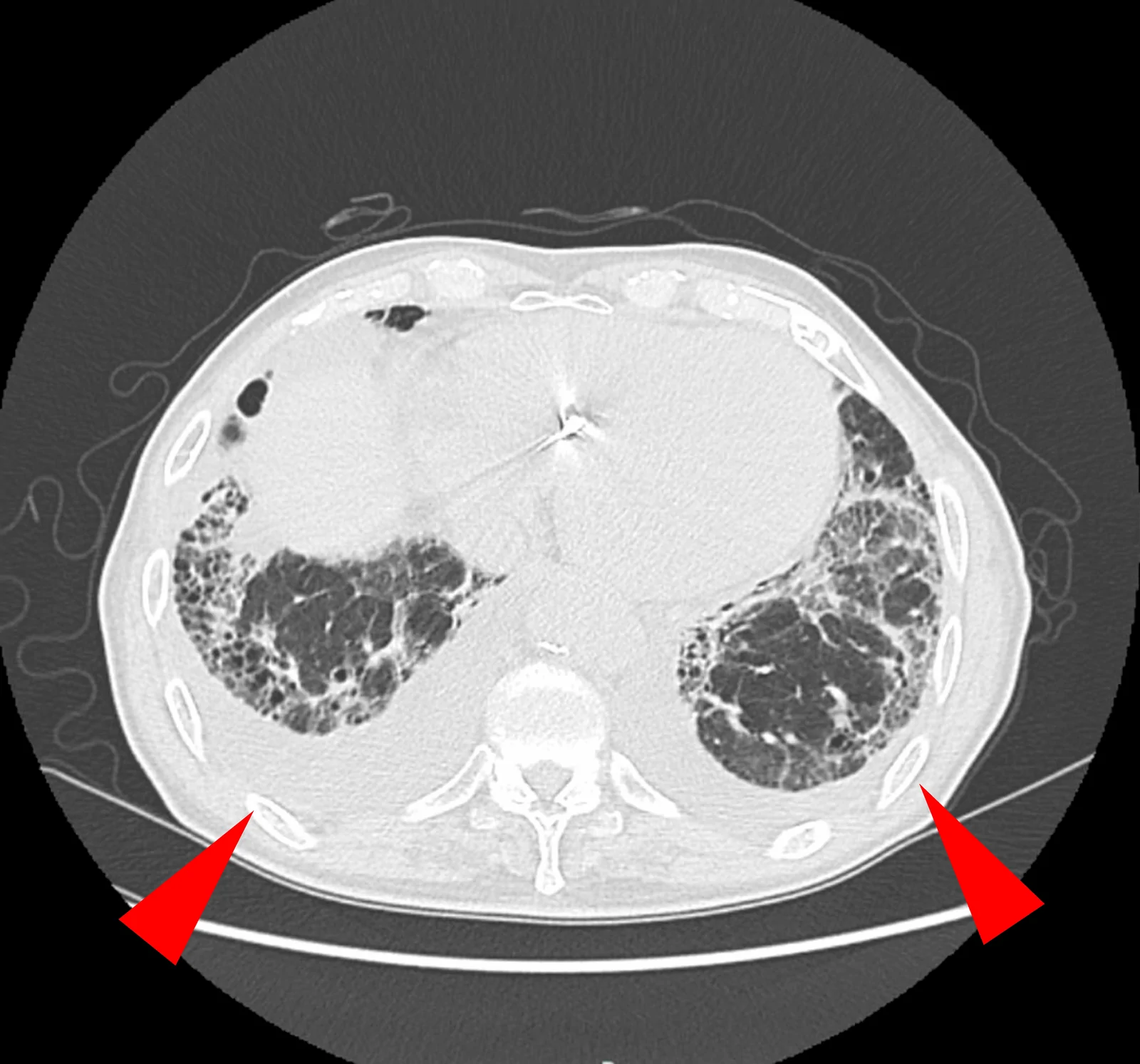
Chest computed tomography on hospital day 5 (one day before respiratory deterioration) Chest computed tomography demonstrating persistent bilateral ground-glass opacities and fibrotic changes with newly developed bilateral pleural effusions, highlighted by red arrowheads

**Figure 4:**
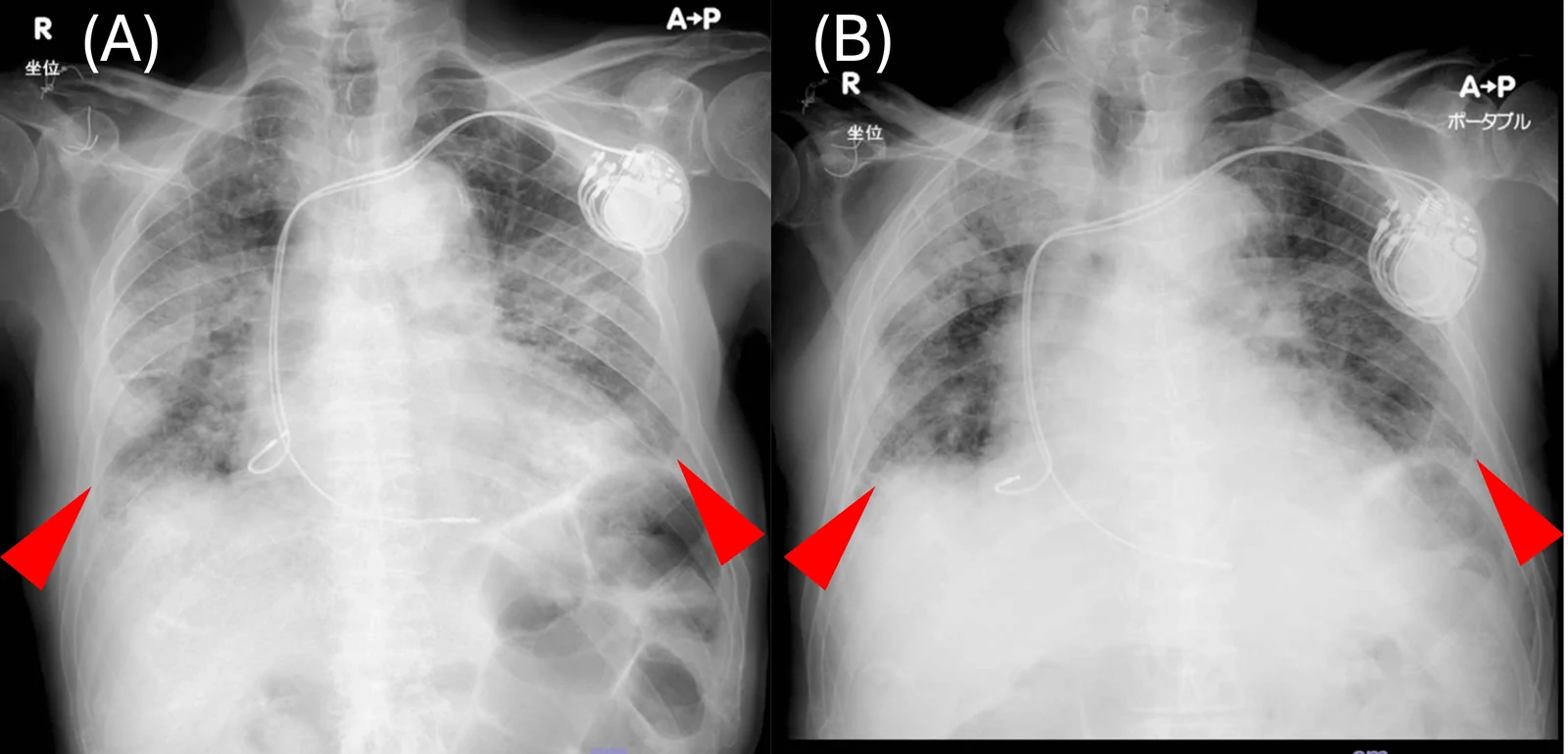
Chest radiographs on admission (A) and hospital day 10 (B) Serial chest radiographs demonstrating increased bilateral pulmonary opacities on hospital day 10 (B) compared with admission (A), highlighted by red arrowheads

After admission, treatment with ceftriaxone 2 g/day and prednisolone 30 mg/day was initiated. His respiratory status initially improved; however, chest CT performed on hospital day 5 showed worsening bilateral ground-glass opacities and bilateral pleural effusions compared with the findings on admission (Figure 3). Because the newly developed bilateral pleural effusions raised concern for worsening heart failure, diuretic therapy was initiated on hospital day 5, resulting in an adequate diuretic response. Nevertheless, on hospital day 6, his hypoxemia rapidly progressed. Arterial blood gas analysis showed a partial pressure of oxygen (PaO₂) of 51.1 mmHg, partial pressure of carbon dioxide (PaCO₂) of 37.3 mmHg, and a P/F (PaO2/FiO2) ratio of 152 while receiving 3 L/min oxygen via nasal cannula. Because of worsening hypoxemia, respiratory support was escalated to high-flow nasal cannula (50 L/min, FiO₂ 40%), improving his oxygen saturation from 88% to 94%. Blood cultures obtained on admission remained negative. Given the worsening bilateral ground-glass opacities superimposed on pre-existing ILD and the continued respiratory deterioration despite diuresis, AE-ILD was clinically diagnosed. Bronchoscopy with bronchoalveolar lavage was not performed because of the patient's advanced age, rapidly progressive respiratory failure, and the risk of respiratory deterioration associated with the procedure. Surgical lung biopsy was also not pursued because the procedural risks were considered to outweigh the potential diagnostic benefit in this clinical setting. Because the patient’s family requested aggressive treatment, methylprednisolone 1,000 mg/day was administered for 3 days, with concomitant administration of cyclophosphamide 500 mg on the first day. After completion of corticosteroid pulse therapy, treatment was changed to prednisolone 50 mg/day. On hospital day 12, prophylaxis against Pneumocystis jirovecii pneumonia with trimethoprim-sulfamethoxazole 1 g once every other day was initiated. Because of persistent disease activity, tacrolimus 4 mg once daily was added on hospital day 15. Chest radiography showed worsening bilateral pulmonary opacities on hospital day 10 compared with those on admission (Figure 4).

## Discussion

This case highlights that delayed healthcare utilization is a multifactorial phenomenon rather than simply a matter of individual patient decision-making [4]. The Andersen Healthcare Utilization Model and the Health Belief Model suggest that healthcare utilization is determined by interactions among patient characteristics, perceived disease severity, barriers to care, and enabling factors, rather than by medical need alone [5,6]. In the present case, despite having respiratory symptoms, there was a delay before the patient was referred to our hospital for further evaluation and treatment. In addition to the possibility that he underestimated the severity of his symptoms, various barriers influencing healthcare-seeking behavior may also have contributed. On the other hand, the patient did visit his primary care physician after symptom onset, indicating that he did not avoid medical care altogether. Initially, he was treated for pneumonia, and although radiological findings suggestive of ILD had been present previously, no continuous follow-up had been provided. In retrospect, the subpleural fibrotic abnormalities identified on chest CT 3 years before admission may have represented clinically significant fibrotic interstitial lung abnormalities that warranted further clinical evaluation and follow-up [3]. Therefore, the delayed access to our hospital in this case may have resulted not only from patient-related factors but also from healthcare provider- and healthcare system-related factors, including the course of the initial management, the timing of referral, and the lack of an appropriate follow-up system. Furthermore, a national survey conducted by the Japanese Ministry of Health, Labour and Welfare found that approximately one-third of individuals with subjective symptoms did not seek medical attention, indicating that healthcare-seeking behavior is not determined solely by the presence of symptoms [4]. Although this patient maintained family relationships, there was little evidence to suggest that social isolation was the primary cause of the delayed healthcare-seeking behavior. Rather, given that he had been treated for pneumonia by his primary care physician, this clinical course may ultimately have influenced the timing of referral to a specialized medical institution. Therefore, interventions aimed at reducing delays in healthcare utilization should extend beyond simply encouraging patients to seek medical attention. A multifaceted approach is required, including shared understanding of the disease between patients and healthcare providers, individualized assessment and reduction of barriers to healthcare utilization, establishment of an appropriate follow-up system, and effective regional collaboration with specialized medical institutions.

AE-ILD is a life-threatening condition associated with high mortality. Although high-dose corticosteroid pulse therapy and immunosuppressive treatment are commonly used in clinical practice, robust evidence supporting these treatments, particularly in the oldest-old population, remains limited. In the present case, worsening radiological findings were observed on chest CT on hospital day 5, followed by rapid respiratory deterioration on hospital day 6. AE-ILD was therefore suspected, and corticosteroid pulse therapy together with cyclophosphamide was initiated. Because of the patient's rapidly progressive respiratory deterioration despite initial antibiotic and corticosteroid therapy, and his preserved pre-admission functional status, aggressive treatment was selected after discussion with the patient's family. Tacrolimus was subsequently added because of persistent disease activity despite corticosteroid pulse therapy and cyclophosphamide, although evidence supporting this strategy is limited. Despite these interventions, the patient ultimately died. Although robust evidence is limited, corticosteroid pulse therapy with or without immunosuppressive agents is commonly used in clinical practice for severe AE-ILD, particularly when rapid disease progression is suspected. A nationwide retrospective study of patients with AE-IPF reported that adding cyclophosphamide to corticosteroid therapy was not associated with reduced in-hospital mortality [7]. Although evidence regarding the treatment of AE-ILD remains limited, improving prognosis through therapeutic intervention after the onset of acute exacerbation alone continues to be challenging. In the present case, the patient's respiratory condition initially improved; however, he subsequently developed rapidly progressive AE-ILD and could not be rescued despite aggressive treatment. In older adults, invasive treatment is often withheld because of advanced age. However, recent studies suggest that frailty and impaired ADL, rather than chronological age itself, are more important prognostic factors. The Clinical Frailty Scale (CFS) has been reported to be associated with mortality in patients with acute exacerbation of ILD [8], suggesting that treatment decisions should not necessarily be restricted based on age alone. Although our patient was 92 years old, he had been independent in ADL before admission. Therefore, corticosteroid pulse therapy and cyclophosphamide were selected based on his functional status rather than chronological age alone. On the other hand, this case also demonstrates that AE-ILD can progress extremely rapidly and become fatal even in oldest-old patients with preserved ADL. In other words, although good functional status may support the consideration of aggressive treatment, it does not necessarily guarantee a favorable prognosis. Therefore, clinicians should not assume that advanced age is associated with slower disease progression. Careful monitoring with early recognition of acute exacerbation and individualized treatment decisions based on the patient's overall clinical condition remain essential, even in very elderly patients.

## Conclusions

We reported the case of a 92-year-old man who had been independent in ADLs before admission and developed AE-ILD following respiratory symptoms. Although he was treated with corticosteroid pulse therapy and cyclophosphamide, he ultimately died. This case suggests that healthcare-seeking behavior is determined not only by individual patient decision-making but also by multiple interacting factors, including patient-related, social, and healthcare system-related factors. It also highlights that in oldest-old patients with AE-ILD, disease severity and treatment eligibility should not be determined solely by chronological age, and that a comprehensive functional assessment, including ADL and frailty, is essential. From a clinical perspective, it is important to recognize and address the cognitive and social barriers underlying delayed healthcare utilization in older adults, while ensuring early recognition of underlying ILD and facilitating timely referral for further evaluation and treatment through appropriate follow-up systems and regional healthcare collaboration. Furthermore, even in oldest-old patients, treatment decisions should not be based on age alone but should instead be individualized according to a comprehensive assessment that includes ADL, frailty, and the patient's overall clinical background. As this is a single case report, these observations are hypothesis-generating and require confirmation in larger studies.
